# Complement, a Therapeutic Target in Diabetic Kidney Disease

**DOI:** 10.3389/fmed.2020.599236

**Published:** 2021-01-21

**Authors:** Kelly Budge, Sergio Dellepiane, Samuel Mon-Wei Yu, Paolo Cravedi

**Affiliations:** Department of Medicine, Icahn School of Medicine at Mount Sinai, New York, NY, United States

**Keywords:** diabetes, diabetic kidney disease, C3a, complement, glomerulosclerosis, MAC, C5a

## Abstract

Currently available treatments of diabetic kidney disease (DKD) remain limited despite improved understanding of DKD pathophysiology. The complement system is a central part of innate immunity, but its dysregulated activation is detrimental and results in systemic diseases with overt inflammation. Growing evidence suggests complement activation in DKD. With existent drugs and clinical success of treating other kidney diseases, complement inhibition has emerged as a potential novel therapy to halt the progression of DKD. This article will review DKD, the complement system's role in diabetic and non-diabetic disease, and the potential benefits of complement targeting therapies especially for DKD patients.

## Introduction

Diabetic kidney disease (DKD) is a common microvascular complication of diabetes mellitus. It is a chronic, progressive disease characterized by kidney function decline through hyperfiltration with or without proteinuria not attributable to concomitant kidney diseases. DKD is the leading cause of both chronic and end-stage kidney disease (CKD and ESKD, respectively) worldwide. In the United States, it affects 25–43% of diabetic patients and is the primary kidney diagnosis in more than 50% of subjects requiring kidney replacement therapy (1, 2). Of note, DKD exponentially increases the risk of cardiovascular morbidity and mortality. Affected patients are >5 times more likely to die of vascular disease rather than start dialysis. In the last 20 years, the incidence of diabetes almost doubled in the US, while the fraction of diabetic patients affected by DKD decreased slightly. Consequently, both DKD incidence and prevalence steadily rose, and the disease is now considered a public health concern (1).

Three classes of drugs are approved by the FDA to treat DKD: angiotensin-converting enzyme inhibitors, angiotensin II receptor antagonists, and the sodium-glucose cotransporter 2 inhibitors (SGLT2i). Other therapeutic interventions include the use of glucagon-like peptide-1 receptor agonists, blood pressure and lipid control, dietetic interventions, and physical activity (3). However, despite the recent and considerable improvements in DKD management, the available therapies can slow but neither stop nor revert disease progression. Increasing evidence demonstrates how DKD is sustained by a complex variety of pathogenic mechanisms, including a wide range of inflammatory processes (4). Currently, most of these findings have not been translated into clinical applications.

The complement system is the main soluble component of the innate immunity, primarily known to facilitate pathogen clearance by lysis and opsonization of target cells. Complement dysregulation is observed in most autoimmune disorders and is the central pathogenic mechanism of various systemic diseases (e.g., hemolytic uremic syndrome and paroxysmal nocturnal hemoglobinuria). As for kidneys, complement activation and deposits are frequently seen in immune-complex mediated glomerular diseases. In the past decade, newly discovered genetic mutations associated with the alternative complement pathway activation have been implicated in several glomerular diseases, alluding to a novel classification of membranoproliferative glomerulonephritis (MPGN) and more importantly, therapeutic options (5). In particular, the success of C5 blockade by eculizumab in atypical HUS (6) has tremendously improved clinical outcomes, and the preliminary data on other glomerular diseases such as ANCA-associated vasculitis (AAV) is also encouraging (7). Furthermore, both mice and human data suggested the role of complement activation in the development of acute and chronic kidney injury. Podocytes and kidney tubular epithelial cells express complement receptors, and their activation may contribute to the progression of DKD (8).

Herein, we will review the available evidence about the pathogenic role of complement activation in DKD progression. Moreover, we will discuss available and experimental drugs able to modulate complement activity to identify novel therapies for DKD.

## Diabetic Kidney Disease

**Figure 1:**
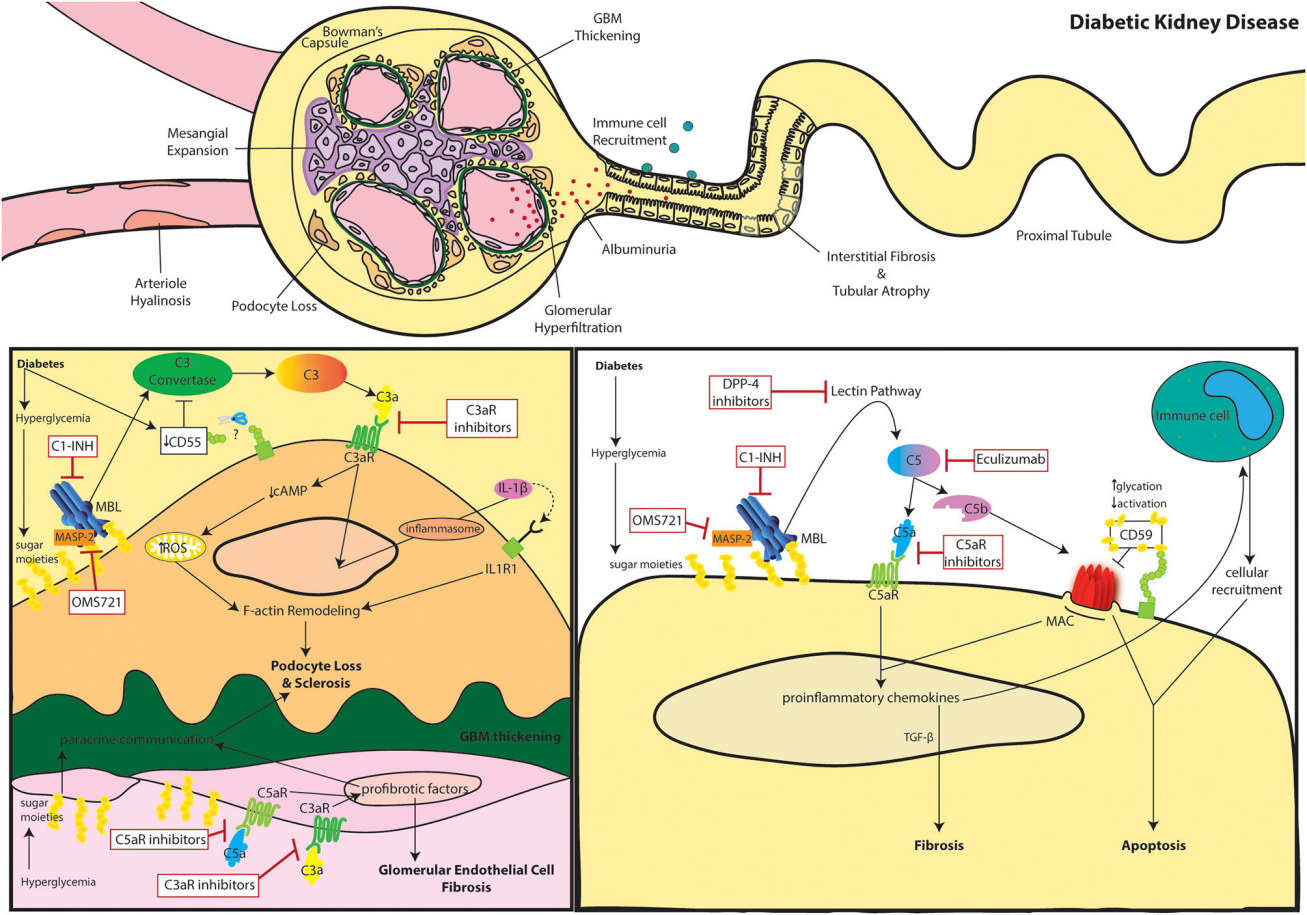
The role of complement in diabetic kidney disease. Clinical manifestations of diabetic kidney disease include arteriole hyalinosis, mesangial expansion, glomerular basement membrane (GBM) thickening, podocyte loss, glomerular hyperfiltration, albuminuria (or proteinuria), immune cell recruitment, interstitial fibrosis, and tubular atrophy (**Top**). Complement activation within podocytes and glomerular endothelial cells is noted in DKD (**Bottom Left**). Diabetes induced downregulation of the C3 convertase inhibitor, DAF, leads to aberrant C3 hydrolysis and subsequent C3a/C3aR signaling in podocytes. Additionally, hyperglycemia induced mannose binding lectin (MBL) activation further propagates the lectin pathway. C3a/C3aR signaling decreases cAMP activity, increasing reactive oxidative species (ROS) production within the mitochondria while similarly inducing IL-1β signaling leading to f-actin remodeling. Cytoskeletal rearrangement eventually leads to podocyte loss and sclerosis after continuous inflammatory insult. Podocyte sclerosis is further promoted by crosstalk with glomerular endothelial cells exposed to hyperglycemia. Paracrine communication of profibrotic factors is promoted by C5a/C5aR and C3a/C3aR signaling similarly leading to fibrosis of glomerular endothelial cells themselves. C3aR and C5aR inhibitors as well as upstream C1-INH and OMS721 complement targets may help relieve glomerulosclerosis. Complement activation within tubular epithelial cells is noted in DKD (**Bottom Right**). Hyperglycemia induces activates complement through enhanced MBL activity. Eventual C5 hydrolysis leads to C5a/C5aR signaling promoting proinflammatory chemokine secretion and TGF-β mediated interstitial fibrosis. The inflammatory response encourages immune cell recruitment leading to cellular infiltrates. The increased glycation events also decrease activation of the membrane attack complex (MAC)-inhibitor, CD59. The combined MAC formation and immune cell infiltration encourage tubular cell lysis and apoptosis. C5aR inhibitors, Eculizumab, DPP-4 inhibitors, C1-INH, and OMS721 may improve disease progression by uncoupling some of these pathogenic mechanisms.

The definition of DKD embraces a variety of pathogenic mechanisms, histological lesions, and clinical manifestations. Generally, DKD is considered a glomerular disease and is classified as a microvascular complication of diabetes. Nevertheless, the whole kidney tissue is involved, and a considerable fraction of patients show significant tubular injury (9, 10). Since the '80s glycemia-induced glomerular hyperfiltration has been considered the main pathogenic mechanism of DKD (11). Hyperfiltration is caused by two distinct phenomena: the enhanced osmolar load of hyperglycemia and the increased sodium reabsorption as a consequence of SGLT-2 hyperactivity. The latter induces a decreased chloride delivery in the macula densa and triggers the tubuloglomerular feedback together with the activation of the renin-angiotensin-aldosterone axis (12). The consequent glomerular disease is characterized by five sequential stages (13): (i) hyper-function and glomerular hypertrophy, (ii) clinically silent stage of morphological lesions (i.e., mesangial expansion), (iii) incipient DKD manifestations with microalbuminuria and elevated blood pressure, (iv) overt kidney disease characterized by persistent proteinuria, decreased kidney function and nodular glomerulosclerosis, and (v) ESKD with diffuse kidney fibrosis. Nonetheless, a minority of DKD patients never manifest a hyper-function phase nor develop proteinuria but still progress to CKD (9). In these patients, other pathogenic mechanisms are thought to lead disease progression which remains unclear. In both subsets of patients, with or without albuminuria, disease progression of DKD is marked histologically by glomerulosclerosis through thickening of the glomerular basement membrane (GBM), mesangial expansion and arteriole hyalinosis (Figure 1).

Hyperglycemia is thought to play an important role in the pathogenesis of diabetic complications. Glycation is the bonding of glucose molecules to proteins or lipids without enzymatic regulation. It causes the accumulation of misfolded and pro-oxidant macromolecules called advanced glycation end-products (AGEs). As a consequence of hyperglycemia, AGEs accumulate in kidney tissues and cause intracellular oxidative stress and extracellular hyaline deposits. Intracellular AGE accumulation activates the transcription factor NFκB (14) in blood mononuclear cells (15). The consequent chronic inflammation induces cell cycle arrest in kidney tubular epithelium and the acquisition of a pro-inflammatory phenotype characterized by cytokine secretion (16). Consistently, intra-kidney inflammation is a key mechanism for DKD progression and is observed in either glomerular or tubular preponderant disease. Single-cell sequencing of glomeruli from a proteinuric DKD mice model demonstrated significant enrichment in activated macrophages (4). On the other hand, in non-proteinuric patients, serum concentrations of markers related to TNF-α and Fas pathways were the strongest predictors of disease progression (17). Evidence in experimental and clinical studies supporting complement's role in kidney injury due to hyperglycemia from diabetes is discussed below.

## The Complement System

**Figure 2:**
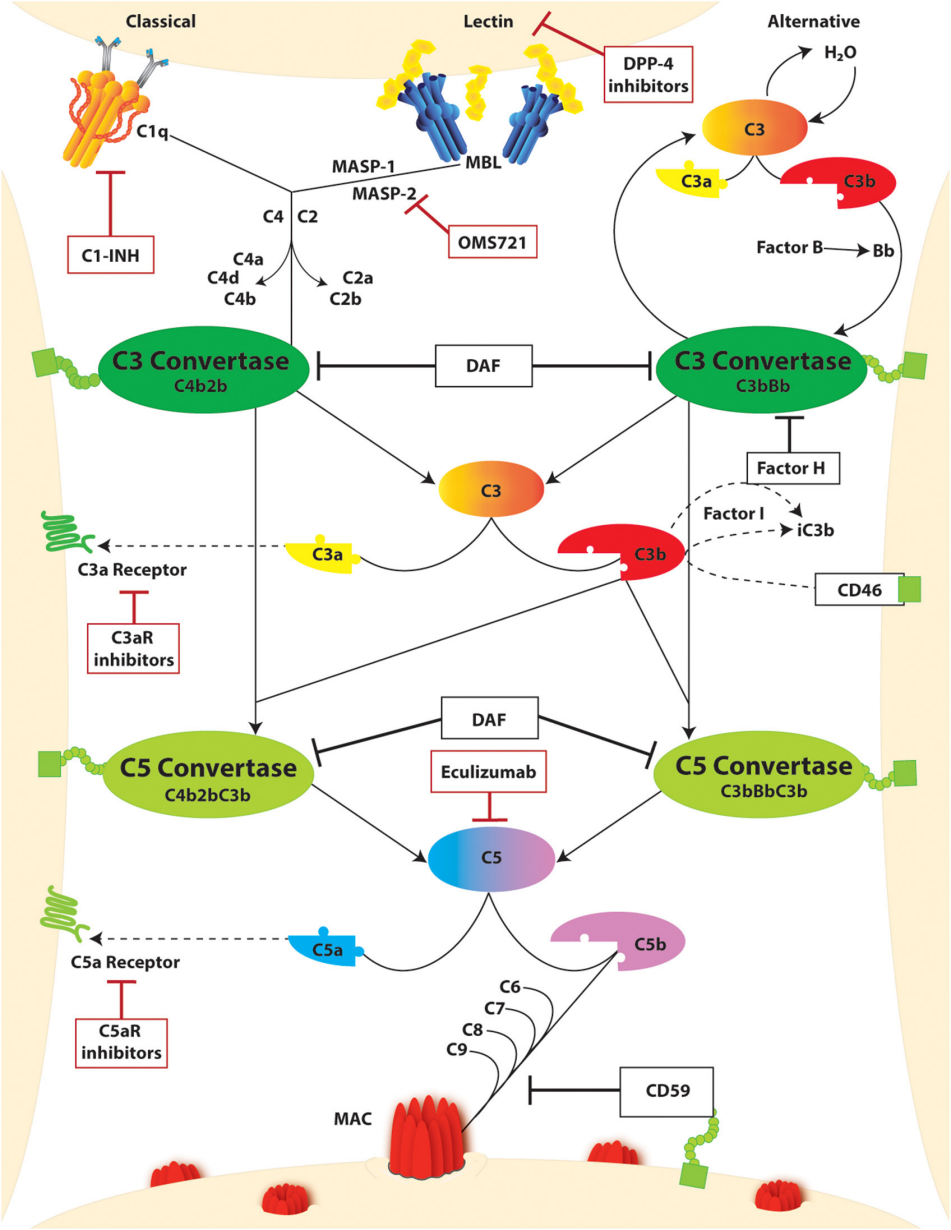
The complement cascade and complement targeting therapies. Activation of the complement cascade occurs through three unique pathways: (i) classical, (ii) lectin or mannose-binding lectin (MBL), and (iii) alternative. Classical and lectin activation through C1q binding to antigen-bound antibodies on the cell surface or MBL binding to bacterial carbohydrate motifs. Respectively, leads to cleavage of C4 and C2 generating the C3 convertase (C4bC2b). In the alternative pathway, complement activation occurs spontaneously through hydrolysis of C3 which binds factor B split product (Bb) to form the C3 convertase (C3bBb). Both C3 convertases cleave C3 yielding C3a and C3b products, the latter of which continues to amplify the cascade. Regulation of this amplification through Factor H and CD46, both in conjunction with Factor I, leads to inactivation of C3b as iC3b. C3b can further bind with C3 Convertases yielding C5 convertases (C4b2bC3b and C3bBbC3b) which cleaves C5 to C5a and C5b. The decay accelerating factor (DAF) competitively inhibits C3 and C5 convertase activity by facilitating disassociation of the assembled enzymes. C3a and C5a split products are signaling molecules which bind the g protein coupled receptors C3aR and C5Ar, respectively. C5b binds to C6-9 to form the membrane attack complex (MAC) which promotes cell injury through pore formation in the cell membrane. This step is inherently regulated by CD59. Further regulation of this pathway through complement-targeted therapeutics (red boxes) include C1-INH, dipeptidyl peptidase-4 (DPP-4), eculizumab, and C3aR and C5aR inhibitors.

The complement system is constituted by soluble and membrane-bound proteins that respond to alarm signals and, through a proteolytic cascade, generate a plethora of immune effectors. Three main triggers that activate the complement cascade include (Figure 2): the binding of C1q to IgG or IgM immune complexes (classical pathway), the continuous and spontaneous C3 hydrolysis (alternative pathway), and the interaction of mannose-binding lectin (MBL) with bacterial glycosylated molecules rich in mannose (lectin pathway). In physiological conditions, the alternative pathway is constantly inhibited by soluble and membrane-bound complement regulators (e.g., complement factor H, complement factor I, and membrane cofactor protein), but it rapidly escalates whenever inflammation or cell injury downregulates these regulators, unbalancing this fine-tuning. While each pathway is unique in its activation, they all converge to C3 cleavage as a central amplification mechanism. Completion of the cascade eventually primes the assembly of the membrane attack complex (MAC). This leads to the lysis of bacteria (lacking complement regulators), and to other cell sublytic injury. Once cleaved, the complement fragments carry out multiple functions beyond the propagation of the cascade and MAC assembly. In particular, C3a and C5a fragments are known as anaphylatoxins, and they are potent immune chemoattractants upon binding of their respective receptors. On the other hand, C1q, C3b, and C4b not only propagate the cascade with their structural and proteolytic roles, but they also opsonize the immune complexes thus promoting pathogen clearance.

Besides these well-established functions, complement receptors are extensively expressed by immune and non-immune cells and exert a variety of pathophysiologic effects (18). Our group demonstrated that C3a/C3aR promotes podocyte injury, activation of both C3a and C5a receptors promote T cell activation and expansion (19), and C5aR is a key regulator of B cell maturation in germinal centers (20). Other authors reported that complement receptors reduce insulin sensitivity in adipocytes (21), prevent memory formation in hippocampal neurons (22), induce mucosal epithelial activation in response to allergens (23), and modulate endothelial function during inflammation (24).

### The Complement System as a Pathogenic Mechanism of Non-diabetic Glomerular Diseases

The ability of the complement system to target specific surfaces whilst distinguishing foreign or altered surfaces from the host requires ample regulation as uncontrolled complement activation could lead to disease. Evidence of such complement dysregulation is noted in many kidney disease types and this changed understanding of disease pathogenesis can improve clinical management. For instance, improved understandings of the alternative pathway in complement significantly revised the classification of membranoproliferative glomerulonephritis (MPGN). Based on the positivity of immunoglobulin (Ig) staining, MGPN now are divided into Ig (+) C3 (+)-MPGN such as infection, autoimmune/rheumatological diseases, or monoclonal gammopathy-mediated GN, and Ig (−) but C3 (+)-MPGN including C3 glomerulonephritis (C3GN) and dense-deposit disease (DDD) (5). Evidence of complement in the disease pathogenesis of other glomerular diseases has also been demonstrated. In human focal segmental glomerulosclerosis (FSGS) kidney biopsy samples, our laboratory recently demonstrated a remarkable downregulation of decay-accelerating factor (DAF, CD55), a complement regulatory protein (see below), together with positive C3d staining in the glomeruli. This is the results of increased DAF cleavage by phospholipase, despite increased DAF mRNA levels. Importantly, we also found an association between urinary C3a with the progression of proteinuria (25). Our data not only established the link between immune dysregulation with FSGS but more importantly, future therapeutic approaches and available drug targets.

Systemic or locally produced complement activation has also been implicated in the disease pathogenesis of antibody-mediated glomerular diseases. Diseases such as membranous nephropathy and lupus nephritis are characterized by complement deposition which is both a biomarker and direct mediator of disease (26). In anti-neutrophil cytoplasm antibody (ANCA) associated vasculitis (AAV), autoantibodies are directed against neutrophil effector proteins, and activated neutrophils release complement precursors and trigger the activation of the cascade (27). C5aR activation seems to be the central pathogenic mechanism, and specific inhibitors have been tested in clinical trials (see below). Due to complement activity in many tissues, the consequences of its dysregulation are extensive, and increasing evidence has suggested the potential clinical relevance of complement pathway intervention.

## The Complement System in Diabetic Kidney Disease

Similar to other non-diabetic kidney diseases, complement activation has been implicated in the pathogenesis of DKD. Proteomic analysis from laser captured microdissection of human kidney biopsies showed increased complement protein in glomeruli from patients with DKD (28). Hyperglycemia is thought to activate the lectin pathway as a result of glycation of pattern recognition molecules (29) and complement regulatory proteins, causing uncontrolled activation of the complement system (30). Formation of the MAC complex is achieved as a downstream result, therefore activating intracellular, pro-inflammatory, and growth factor signaling pathways in podocytes and other glomerular cells. Extensive crosstalk exists between the resident cells of the glomerular filtration barrier (GFB), in particular podocytes and glomerular endothelial cells, which has been recorded under high glucose conditions and is integral to GFB integrity (31). The diabetic milieu surrounding the glomerulus encourages foot process effacement and detachment of podocytes from the GBM, and it is unclear if this is the result of a primary injury of the podocyte or is driven by abnormalities in glomerular endothelial cells (32). Indeed, high glucose induces morphological changes also in glomerular endothelial cells which is ameliorated by complement receptor (C3aR and C5aR) inhibition both *in vitro* and *in vivo* (33). Tubular deposition of C5a has also been shown to correlate with the severity of human DKD and blocking C5a-C5a1R axis ameliorated interstitial fibrosis in a murine diabetic (*db/db*) model (34), suggesting that complement activation is mediating also tubulointerstitial injury in DKD.

Evidence of glycated complement components was noted in DKD patients over three decades ago (35); glycated C3 and C4 appear early in diabetic patients but their function is not impaired (36). Conversely, the glycation of complement regulator CD59 leads to loss of its MAC-inhibitory function and associates with endothelial cell damage (37). Gosh et al. measured soluble glycated CD59 in a cohort of 500 subjects with or without diabetes (38). Glycated CD59 was significantly associated with other markers of disease progression and substantially decreased after glycemia optimization. However, more studies are warranted to investigate glycated CD59's association with DKD patient outcomes. Lastly, a recent study identified a unique inflammatory signature in a cohort human DKD patients who progressed to ESKD. Of note, DAF was shown to correlate with other inflammatory markers, suggesting possible complement activations in chronic inflammatory milieu of DKD (39).

### Animal Models

Animal models of diabetes have illuminated a deeper understanding of the potential role for the complement system in disease pathogenesis [See (40) for an extensive review; Table 1]. These preclinical studies reported an association between intrarenal C3 deposition and DKD in both T1D (45, 46) and T2D models (47). Intriguingly, C3 deposition is reversed by transplanting kidneys from diabetic mice into euglycemic ones (41). In genetically obese (ob/ob) mice, intrarenal C3a increased in concomitance with DKD development, and C3a/C3aR signaling in podocytes caused cytoskeletal rearrangement and mitochondrial dysfunction leading to podocyte loss and proteinuria (24). Transcriptomic, lipidomic, and metabolic analysis of streptozotocin-induced diabetic mice also observed changes in mitochondrial agility that was restored after inhibition of C5aR (34). Therefore, both C5aR and C3aR propose an attractive target for renoprotection in diabetes. We hypothesized that DKD is associated with glomerular cleavage of the DAF, a key inhibitor of C3 convertase (25). After diabetes induction by streptozotocin, DAF deficient mice have augmented C3b glomerular deposition and manifested a more severe disease phenotype and aggravated histological lesions when compared to control diabetic wild-type mice (Figure 1).

**Table 1:** Murine models of diabetic nephropathy with documented complement activation.

**Model**	**Induction**	**Diabetes type**	**Model description**	**Signs of complement activation**	
Streptozotocin (STZ)	Drug	1	Induction of hyperglycemia through STZ injury to the beta cells of the pancreas	(33)	C3aR and C5aR of glomerular endothelial cells
				(41)	C3 deposition in mesangium reversed in transplant
				(25)	DAF reduction in podocytes
				(42–44)	Lectin pathway involvement (MBL)
NOD	Genetic	1	Autoimmune destruction of beta cells	(45)	C3 deposition in glomeruli
OVE26	Genetic	1	Transgene that increases calmodulin expression in the beta cells of the pancreas	(46)	C3 gene expression in tubules
ob/ob	Genetic	2	Mutation in leptin that causes obesity induced hyperglycemia	(24)	C3/C3aR signaling in podocytes
db/db	Genetic	2	Mutation in the leptin receptor that causes obesity-induced hyperglycemia	(34)	C5a deposition in tubules
KK-Ay	Genetic	2	Mutation in nonagouti (A^y^) that casues obesity-induced hyperglycemia	(47)	C3 deposition in glomeruli and glomerular capillaries

Preclinical data have indicated the relevance of the lectin pathway in the pathogenesis of DKD. In the streptozotocin-induced diabetes model, MBL-knockout mice had reduced kidney weight, urinary albumin excretion, and kidney fibrosis when compared to wild-type diabetic animals (42). Other animal studies using this model demonstrated that MBL half-life is increased after diabetes onset (43) and that MBL accumulates within the glomerular tissue (44). The altered cell surface in the glomeruli due to hyperglycemia are likely to recognized by MBL, and MBL autoreactivity may play a role in complement activation and DKD pathophysiology.

While these models provide valuable insight for preclinical studies, there are limitations to these animal studies. DKD progression in humans requires many years, whereas many animal studies are limited to weeks or months. Therefore, it is necessary to validate these animal findings in human studies.

### Human Data

While complement activity is noted in diabetes, complement activation is particularly evident when kidney disease occurs. In a gene-expression analysis of postmortem kidneys, complement was mostly altered in early DKD when only limited glomerulosclerosis was evident (48). Also, compared to diabetic subjects without kidney disease, DKD patients have significantly higher levels of MBL, Bb, C4d, C3a, C5a, and soluble MAC in both plasma and urine (49). Of note, most of these factors are overexpressed within the kidney parenchyma of diabetic rats and were associated with intra-renal MAC generation (50).

Multiple clinical studies reported hyper-activation of the lectin pathway in DKD similar to the animal studies mentioned above. In 191 T1D patients from the Finnish Diabetic Nephropathy Study, MBL levels were 50% higher in those affected by proteinuria and significantly correlated with Hb1ac and kidney glucose disposal rate (51). In a cohort of 326 T2D patients followed for 15 years, higher MBL values were associated with a 1.5 hazard ratio for death and 2.6 for the development of proteinuric kidney disease (52). The same group published data from 1,564 T1D patients with a median follow-up of 5.8 years; MBL values significantly correlated with urinary albumin levels and predicted the onset of end-stage kidney disease (53). Zheng et al. studied 62 kidney biopsies from DKD patients and observed prominent MBL, MBL-associated serine protease 1 (MASP1), and MAC immune-reactivity in tubulointerstitial tissue; by converse, intrarenal C1q deposits did not correlate with disease status, suggesting that the classical pathway of complement activation does not play a dominant role (29). Consistently, another protein able to activate the lectin pathway, H-ficolin, predicted microalbuminuria development in incident T1D patients followed for 18 years (54). Additionally, genetic studies were able to identify multiple MBL polymorphisms that associate with DKD onset and progression (53, 55). This data combined with the preclinical studies indicate the putative mechanism of MBL activation is the cross-reactivity of the mannose-binding domain with AGEs as demonstrated by multiple binding assays (56).

Downstream complement products have been associated with DKD activity as well. Urinary excretion of C3b, Bb, and MAC have been noted in DKD patients (57) and more recent evidence indicates that the presence of complement split products in the urine is associated with accelerated ESKD and death (58). Although the timing of complement activation in the development of DKD remains unclear, urinary complement products do correlate with tubular interstitial injury, supporting a major pathogenic role for complement activation in DKD progression (59). Transcriptomic analysis of advanced DKD glomeruli and tubules have also indicated differential expression of the complement pathway regulatory proteins and products. Canonical pathway analysis noted that within the complement system, C3, CD55, C1QA, CD46, C1QB, CFB, C4A/C4B, C7, CFH, C3AR1, CR1, and C2 in DKD glomeruli showed a three-fold increase in their expression compared to healthy controls indicating complement is likely locally synthesized within the DKD glomerulus (60). While the upregulation of complement regulatory transcripts may seem to contradict a previously noted downregulation of this protein in non-diabetic glomerular disease leading to uninhibited complement activation, we similarly saw an increase in CD55 mRNA in FSGS patients but a decrease in transmembrane CD55 staining in FSGS biopsies. This decrease in protein is likely due to cleavage of CD55 that can be found in the urine of Adriamycin treated mice and FSGS patients (25). A similar mechanism could be occurring in DKD. It may also be argued that this advanced-stage of DKD is associated with glomerulosclerosis which could indicate non-specific complement activation, but differential transcriptomics of early-stage DKD, prior to proteinuria or changes in eGFR, have also indicated dysregulated complement expression to be the most significantly altered canonical pathway (48). Specifically, C7 expression and gene product were notably upregulated in early-stage DKD which was suggested as an early-detection therapeutic for otherwise indiscernible renal damage (48).

## Complement Targeting Therapies Evaluated in Kidney Disorders: A Possible Horizon for DKD

Complement activation is a primary pathogenic mechanism in numerous inflammatory disorders and substantial efforts have been made to develop complement-targeting drugs. In March 2007, Eculizumab became the first complement-specific drug being approved by the FDA for the treatment of paroxysmal nocturnal hemoglobinuria and, subsequently, aHUS (61). Nowadays only one other agent (C1-inhibitor or C1-INH) has reached FDA approval (62), but other molecules are under investigation [reviewed by (63)].

### C3aR and C5aR Inhibitors

Growing evidence has unveiled the primary role of complement receptors in DKD. Morigi et al. (64) showed that C3aR antagonism preserved podocyte number and prevented both proteinuria and kidney function decline in the ob/ob murine model of DKD (monogenic obesity). Similarly, a C5aR inhibitor (K-76 COONa) reduced DKD severity and glomerular lesions in rats (65). Complement components that were bound to the untreated diabetic rats' injured glomeruli were not observed in the treatment group indicating complement activation exacerbated diabetic glomerulosclerosis. Complement receptor blockade in diabetes may also target extrarenal disease mechanisms. In obese rats, both C3aR and C5aR antagonists reduced the expression of proinflammatory adipokines and other inflammatory genes in the adipose tissue (21). The wide capacity of these antagonists thus implicates an imaginable holistic therapeutic from metabolic and nephrotic approaches.

While animal models of DKD invite promise for the use of C3aR and C5aR inhibitors, the study of these inhibitors in humans is limited. A C5aR inhibitor (CCX168) has been branded by ChemoCyntryx as Avacopan and released for experimental human use (66). In ANCA-associated vasculitis, CCX168 alone or in combination with low-dose corticosteroids was non-inferior to standard high-dose corticosteroid treatment (67). The drug is currently under investigation as a treatment option for hidradenitis suppurativa (NCT03852472), C3 glomerulopathy (NCT03301467), hemolytic uremic syndrome (NCT02464891) and IgA nephropathy (68). More data about the long-term safety of CCX168 are needed to experiment with this drug in DKD.

### C1-INH

C1-INH is the recombinant form of human C1 esterase inhibitor and is commercially available as Cinryze (Shire pharmaceutics). C1-INH is a complement regulating protein that halts the classical pathway and regulates the intrinsic coagulation cascade. It is currently FDA-approved for the treatment of hereditary angioedema (i.e., the genetic deficiency in C1-INH) (62). A phase II randomized and placebo-controlled trial investigated the use of C1-INH in 70 patients at high risk of delayed graft function (DGF) after kidney transplantation (69). The study did not meet the primary endpoint (reduction of DGF incidence), but treated patients required fewer dialysis sessions and had a higher glomerular filtration rate at 1-year follow-up. Similarly, a pilot study about antibody-mediated glomerular damage post kidney transplantation showed a trend toward kidney function improvement and reduced glomerular damage (70). MBL and C1q share relevant structural and functional homologies (71); in particular, both activate C4 and C2 to generate the C3 convertase C4bC2a (72) and both are inhibited by C1-INH. As such, C1-INH could downregulate the lectin pathway in DKD and may represent a new therapeutic approach. Clermond et al. observed a reduction of diabetic retinopathy in rats treated with intravitreal C1-INH. Of note, DKD and diabetic retinopathy have largely overlapping pathogenesis; however, the authors ascribed this effect to the inhibition of plasma kallikrein, another target of C1-INH (73).

### Eculizumab

Eculizumab is a murine humanized monoclonal antibody that binds with high-affinity C5 and prevents its cleavage and the generation of C5a and MAC. It is commercialized by Alexion Pharmaceuticals as Soliris. Eculizumab was sequentially approved for the treatment of paroxysmal nocturnal hemoglobinuria, atypical hemolytic uremic syndrome, and neuromyelitis optica in adults who are anti-aquaporin-4 antibody positive.

Eculizumab has also been used with promising results to treat C3 glomerulopathies (74, 75) and antibody-mediated kidney transplant rejection (76, 77). It has also been tested, but with no success, to prevent delayed kidney graft function (DGF) after transplantation (78). Several clinical studies are ongoing for other conditions, including HELLP syndrome (hemolysis, elevated liver enzymes, low platelet count - NCT04103489), and Guillain-Barre syndrome (NCT02029378). No data are available on eculizumab in DKD. However, considered the large volume of patients treated, a well-designed case-control study on diabetic individuals who received eculizumab for other reasons may provide relevant insights about C5 inhibition in DKD.

### Lectin Pathway Targeting

OMS721 is a monoclonal antibody targeting MBL-associated serine protease-2 (MASP-2), the protease that binds MBL and cleaves C4 and C2. OMS721 is currently under investigation for TMA, aHUS (NCT02222545), and IgA nephropathy (NCT02682407). Given the specificity for the lectin pathway, this agent could represent a valid option for DKD without inducing broad complement inhibition.

### Dipeptidyl Peptidase-4 as Possible Complement Modulators

Dipeptidyl peptidase-4 (DPP-4) is a serine protease that inactivates glucagon-like peptides and is targeted by a widely used anti-diabetic drug class (DPP-4 inhibitors). Hoffman-Petersen and colleagues (79) demonstrated *in vitro* that DPP-4 inhibitors block also complement-activating serine proteases and inhibit in particular the lectin pathway. The authors hypothesized that DPP-4 reduce complement injury in DKD and randomly assigned 137 patients to sitagliptin or placebo. Sitagliptin-treated patients had reduced circulating levels of MBL and soluble MAC after 12 weeks of treatment.

### Glycated CD59

As previously noted, hyperglycemia can cause glycation of CD59 which normally inhibits MAC complex formation (37). Glycation-induced inactivation of CD59 leads to enhanced complement activity and is involved in the pathogenesis of hyperglycemia mediated end-organ complications. Effective screening for comorbidities such as early gestational diabetes mellitus and large for gestational age newborns has already been shown when glycated CD59 levels were measured (80). In addition to the use of glycated CD59 as a biomarker for DKD, the upregulation of CD59 presents a potential mechanism for inhibiting the terminal pathway of complement activation. In neurons, RE1-silencing transcription factor (REST) targeted the upregulation of CD59 protected against complement-mediated lysis (81). Further mechanistic understanding of CD59 inactivation in DKD may elucidate further complement targeted therapeutics.

## Conclusion

Diabetic kidney disease is a frequent complication of diabetes and the leading cause of chronic kidney disease worldwide. In recent years, new drugs have considerably improved DKD management by targeting glucose metabolism; however, different disease mechanisms may promote DKD progression once the initial injury is established. The complement system is hyper-activated in DKD and has been recognized as the main mechanism of disease progression. In particular, AGEs trigger the lectin pathway, whose activation significantly associates with patient prognosis. Besides MAC generation, the complement cascade triggers kidney injury through complement receptors expressed on kidney cells. Pre-clinical studies demonstrated that complement inhibition prevents DKD progression and complement receptor targeting is a promising therapeutic strategy.

## Author Contributions

KB, SD, and SY wrote the manuscript. PC supervised the manuscript. All authors contributed to the article and approved the submitted version.

## Conflict of Interest

The authors declare that the research was conducted in the absence of any commercial or financial relationships that could be construed as a potential conflict of interest.
